# Strengthening vaccine capacity building on the African continent

**DOI:** 10.1038/s41467-025-62839-y

**Published:** 2025-08-13

**Authors:** Nicaise Ndembi, Jerome H. Kim

**Affiliations:** 1 International Vaccine Institute (IVI), Norrsken House, 1 KN 78 St, Kigali, Rwanda; 2 International Vaccine Institute (IVI), College of Natural Sciences, Seoul Natural University, Seoul, Republic of Korea

## Abstract

The African Union (AU) has set an ambitious goal to strengthen primary health care systems on the African continent and achieve universal health coverage for all African citizens by 2030. As part of this initiative, the AU advocates for African self-reliance in the manufacturing of life-saving vaccines and drugs. Here, we speak with Professor Nicaise Ndembi and Dr Jerome Kim, who are at the forefront of capacity building for African vaccine manufacturing, about the progress that has been made towards the AU’s goal, and what challenges remain.

**Figure:**
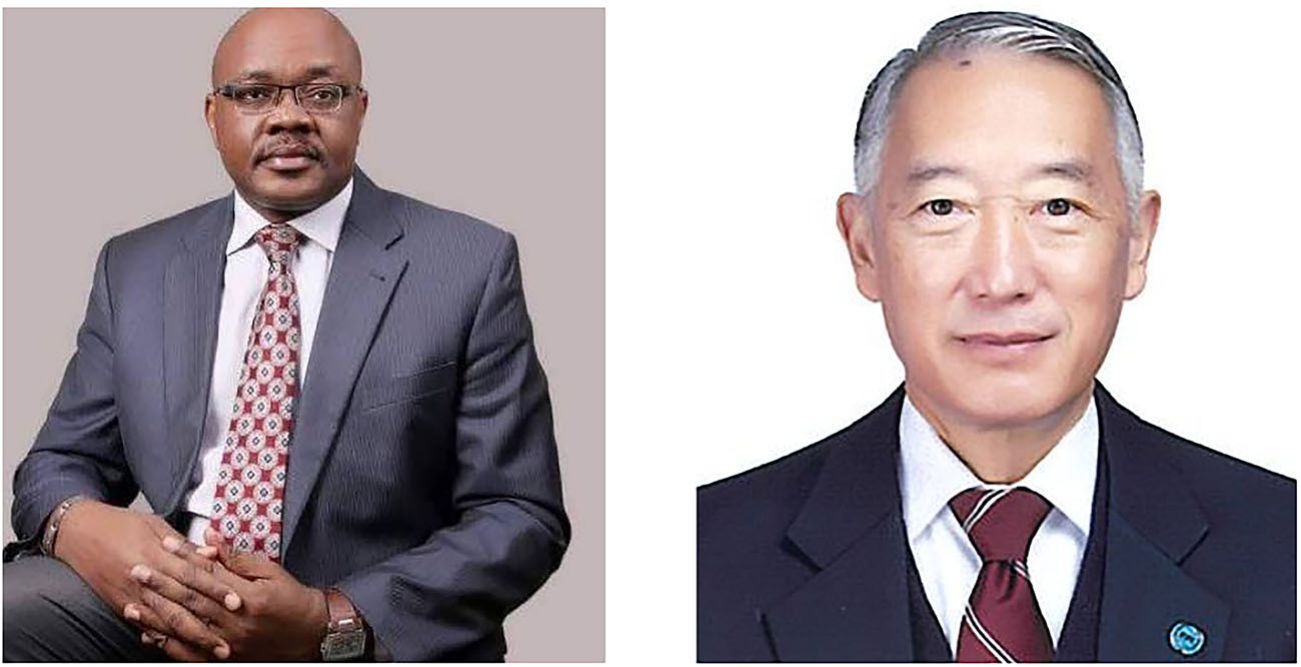
Professor Nicaise Ndembi (Left) and Dr Jerome Kim (Right).

The African Union’s Roadmap to 2030 & Beyond comprised ambitious commitments to strengthen primary health care systems on the African continent and to achieve universal health coverage for African citizens. Please, would you elaborate on the overall goals of the roadmap?

The African Union Roadmap to 2030 is part of its agenda to transform the African continent into a global powerhouse by 2063^1^. As part of its 2063 Agenda, the African Union recognizes health as a foundational enabler of sustainable development. The burden of diseases, including HIV, tuberculosis, malaria, as well as emerging infectious threats, continues to undermine Africa’s productivity, education, and economic growth; the Roadmap to 2030 proposes a holistic approach to address these challenges by linking socioeconomic development with health security, through universal health coverage (UHC) and resilient health systems, for all Africans.

The AU Roadmap to 2030 outlines seven strategic pillars (Table 1), which each represent a critical building block towards resilient, inclusive, and self-sufficient health ecosystems^2^. The pillars outline a bold, integrated strategy to achieve health security and UHC across Africa through building resilient health systems capable of managing threats like epidemics and climate-sensitive diseases; ensuring equitable access to quality healthcare without financial hardship; transforming primary healthcare into a community-owned, people-centred foundation; promoting self-reliance through local manufacturing and financing; and ensuring sustainability via strong governance, community ownership, and a skilled health workforce. The roadmap devolves decision-making, builds regional capacity, reduces external dependency, and designs equitable, sustainable health systems rooted in African realities.

**Table 1:** The seven strategic pillars of the African Union Roadmap to 2030 for improved health security for all African citizens

Pillars	Intention
Pillar 1: Adolescents, Children, Men, Women and Youth	Engage communities in health initiatives by promoting awareness, and fostering ownership of health outcomes
Pillar 2: Health Equity and Vulnerable Populations	Ensure that prevention, treatment, and care services are accessible to every individual, particularly marginalized and vulnerable populations
Pillar 7: Service Delivery for HIV, TB and Malaria, NTDS, STIs and Viral Hepatitis, NCDs and RMNCAH	
Pillar 3: Access to medicines, regulatory harmonization and local/regional manufacturing of medicines, vaccines, and diagnostics	Enhance healthcare infrastructure and workforce capacity to provide quality services for all through strategic partnerships
Pillar 4: Health Security and Health Systems Strengthening	
Pillar 5: Diversified and Sustainable Financing	Promote shared responsibility and global solidarity by developing innovative financing mechanisms and mobilizing both domestic and international resources
Pillar 6: Leadership, Governance, Community Engagement and Oversight for Sustainability	Integrate health initiatives into broader development goals to create resilient communities capable of addressing current and future health challenges

What successes and lessons have been learned since the rollout of the AU Roadmap to 2030?

During the peak of the COVID-19 pandemic, the Africa Centres for Disease Control and Prevention (Africa CDC) was instrumental in coordinating regional efforts to mitigate the impact of the virus across the continent. Africa CDC significantly expanded testing capacity through the Partnership to Accelerate COVID-19 Testing (PACT), distributing over 10 million test kits to enable earlier detection and response. The African Pathogen Genomic Initiative strengthened surveillance by building genomic sequencing capacity in multiple countries, allowing real-time tracking of variants to guide targeted public health actions. To support safe cross-border movement and economic recovery, the African Union Trusted Travel Portal provided a centralized platform to verify test results and vaccination status. The African Union COVID-19 Response Fund mobilized critical resources that empowered local health systems to procure essential equipment and bolster frontline response. Demonstrating local ownership, the African Vaccine Acquisition Trust (AVAT) pooled resources to negotiate vaccine procurement directly with manufacturers, ensuring equitable access beyond reliance on donations. The Africa CDC Consortium for COVID-19 Vaccine Clinical Trials (CONCVACT) accelerated vaccine research by coordinating trials across several African sites, strengthening local scientific leadership, and generating relevant data. In addition, the Africa Medical Supplies Platform (AMSP) streamlined procurement and distribution of essential supplies, improving supply chain transparency and responsiveness. Together, these initiatives show how the Africa CDC turned strategic goals into concrete actions that enhanced health security, surveillance, and local leadership in managing the pandemic^3^.

On a continental level, and outside of the height of the COVID-19 pandemic, the MenAfriVac vaccine against *Neisseria meningitidis* serogroup A showcases effective African health collaboration by targeting children and adults aged 1–29 years in the 26 countries of Africa’s meningitis belt^4^. Its phased rollout began with mass campaigns from 2010 to 2016, vaccinating over 300 million people, and later integrated into routine immunization for one-year-olds. Focusing on meningitis belt countries, coverage exceeded 90% in targeted areas by 2013, leading to a > 99% decline in meningitis A cases by 2020^5^. Despite initial exclusion of pregnant women and delays in conflict zones, success was driven by community engagement, political will, regional coordination, and local manufacturing partnerships, including tech transfer to the Serum Institute of India, which ensured affordable doses (~$0.50)^6^. This experience aligns with AU Roadmap pillars on local production and community involvement and guides current vaccine manufacturing efforts by the Africa CDC and partners.

At a country-wide level, Rwanda’s Mutuelle de Santé achieved 88% population coverage by 2022, slashing out-of-pocket health expenses from 28% to 12% of total health expenditure between 2003 and 2014, and enabling near-universal access to primary care^7^. This serves as a model for domestic financing (Pillar 5) and equity (Pillar 2). Also, Ethiopia’s Health Extension Program, deploying >40,000 trained community health workers, had a 37% decline in the maternal mortality rate attributed to this program, with approximately 40,000 deaths prevented between 2015–2020^8^, demonstrating how workforce expansion (Pillar 4) and community engagement (Pillar 6) save lives.

Despite these examples, country-level successes are not uniform across the continent; countries including Nigeria and Kenya continue to struggle with fragmented health service delivery, inconsistent healthcare funding, and gaps in health infrastructure, particularly in rural and underserved areas. These disparities underscore the need for coordinated investment, domestic resource mobilization, sustained long-term political will, and systemic-level reform. Much of the success of the programs in Rwanda and Ethiopia is due to community trust and participation in driving uptake and compliance, demonstrating the critical role that people-centred partnerships have for progress towards the goals of the AU Roadmap. In addition, there remains a need for building resilient primary healthcare systems, facilitating data-driven decision-making, and developing a local and trained health workforce; all of these require technical solutions, political commitment, community engagement, and sustainable resourcing.

The African Union has set a goal for the African continent to produce over 60% of its vaccine needs by 2040. What are the reasons behind this goal?

The COVID-19 pandemic exposed Africa’s heavy reliance on external suppliers as African countries received vaccines much later than high-income nations; while some countries began vaccination in late 2020, many African nations had to wait until mid-2021 to access limited doses, largely through donations or the COVAX facility. This inequity underscored the vulnerability of Africa’s health systems and the urgent need for manufacturing independence. More recently, Africa’s vaccine response to the mpox outbreaks was delayed due to its reliance on importing vaccines.

Recognizing the importance of strengthening regional health security and self-reliance, African Heads of State called for increased local production of life-saving vaccines, and the African Union set a continental goal to meet 60% of Africa’s vaccine demand with locally manufactured products by 2040^9^. Local production would reduce costs, shorten supply chains, improve timely access to life-saving vaccines, and enhance the ability to respond swiftly to disease outbreaks. Expanding vaccine production in Africa also presents an opportunity to boost economic development, create high-skilled jobs, and foster innovation.

There are two key steps in the vaccine manufacturing process, which are the production of the antigen and the formulation of the final vaccine (form/fill/finish). In 2023, Africa CDC, PATH and the Clinton Health Access Initiative published a joint assessment of current vaccine manufacturing capabilities on the African continent; one of the central findings was that African vaccine manufacturing capacity is concentrated on form/fill/finish and this could be readily scaled up. What progress has been made towards this goal?

The form/fill/finish stage of vaccine manufacturing is the final stage in which the syringe is filled, labelled, packaged, and inspected for quality. It is the last stage before the vaccine leaves the manufacturer. If this process was scaled up on the African continent, it would mean shorter supply chains and the rapid deployment of life-saving vaccines. Since 2023, Africa has made significant progress in laying the groundwork for local/continental vaccine manufacturing (Table 2).

**Table 2:** Selected successes in capacity building and vaccine manufacturing on the African continent since 2023

Country	Institute	Success
Senegal	Pasteur de Dakar	High-capacity fill-finish facility opened in 2023 with support from the IFC and European Partners
South Africa	Biovac	Expanding its oral cholera vaccine manufacturing in partnership with the International Vaccines Institute (IVI). This would be the second drug substance (DS) manufacturing in Africa.
South Africa	Afrigen Biologics	Recreated Moderna’s mRNA COVID-19 vaccine using publicly available sequences. This was made possible through the World Health Organization mRNA technology transfer and Medicines Patent Pool programme with support from the international donors.
South Africa	Aspen Pharmacare	Fill&Finish of COVID-19 vaccine. This has been made possible with support from the Wellcome Trust.
South Africa	Hexaxim	Local manufacturing of this combination vaccine that provides immunization against six pediatric diseases: diphtheria (D), tetanus (T), pertussis (whooping cough), polio, hepatitis B (HB), and invasive diseases caused by Haemophilus influenzae type b (Hib). This has been made possible with support from the Wellcome Trust.
Rwanda	African Medicines Agency (AMA)	Launched in 2023 to improve the regulation of medicinal products on the African continent. Appointed an inaugural Director General of AMA.

Are there any specific goals to increase Africa’s capacity to produce antigens? What role do technology transfers have in aiding or preventing this goal?

Producing antigens, the biologically active components of vaccines that trigger the immune response, is fundamental to true vaccine self-reliance. Without this capability, Africa remains dependent on importing these core materials, creating vulnerabilities during health emergencies (as starkly demonstrated by COVID-19 vaccine inequity) and limiting the ability to rapidly develop tailored vaccines for continent-endemic diseases like malaria, tuberculosis, HIV, and cholera. Therefore, specific goals exist to significantly increase Africa’s capacity for antigen manufacturing, moving beyond the current focus on final formulation/fill/finish (FFF), as part of the AU and Africa CDC’s broader ambition for vaccine sovereignty. This requires substantial investment in upstream production capabilities, the most technologically complex and capital-intensive part of the vaccine value chain.

Technology transfer is pivotal for achieving this goal. It enables African manufacturers to acquire the knowledge, production techniques, and quality control standards needed for large-scale antigen production. Initiatives like the WHO mRNA technology transfer hub in South Africa, which enabled the local production of a COVID-19 mRNA vaccine candidate, provide crucial blueprints. Success hinges on voluntary licensing agreements, open-source platforms, and structured mentorship to build a critical mass of trained scientists, regulatory experts, and engineers, alongside establishing continental centers of excellence for bioproduction and translational research.

Advancing Africa’s health security requires concerted global action. Originator companies, multilateral organizations (WHO, Gavi the Vaccine Alliance, CEPI, UNICEF), donor agencies, and high-income nations have both a moral obligation and a strategic interest. These stakeholders must evolve beyond short-term grants to become true ecosystem enablers. This involves providing technical mentorship for regulatory harmonization, facilitating pooled procurement to de-risk demand, supporting regional testing labs and FFF facilities, and promoting quality standard convergence. Crucially, global solidarity is needed: relaxing export restrictions, easing IP barriers (including pandemic-era patent waivers), ensuring prioritized access to raw materials, and expediting export clearance for biomanufacturing equipment. Furthermore, global financial institutions must offer tailored funding (infrastructure grants, concessional loans, long-term working capital) for African manufacturers meeting quality benchmarks.

Nicaise, in your previous role, you established the Partnerships for African Vaccine Manufacturing, which is a framework for regional vaccine manufacturing and self-reliance. How did this initiative aid regional cooperation and facilitate capacity building?

The Partnerships for African Vaccine Manufacturing (PAVM) fostered regional cooperation by harmonizing strategy across 55 AU member states, creating a unified roadmap for local production. Crucially, it facilitated capacity building through two key successes. PAVM accelerated the ratification of the AMA treaty, enabling continent-wide regulatory harmonization. This will allow manufacturers like South Africa’s Aspen to distribute COVID-19 vaccines to partner African countries without redundant approvals, boosting cross-border supply chains. In addition, PAVM secured AU backing for the WHO-supported hub in South Africa. This initiative trained 15+ scientists from Nigeria, Kenya, and Senegal in mRNA production, with Senegal now establishing its fill-and-finish facility using transferred expertise. These outcomes directly strengthened regional self-reliance by aligning policy and scaling workforce capabilities across borders.

Despite these gains, critical challenges persist. Infrastructure remains the foremost hurdle, with an estimated $10 billion being required to develop and equip vaccine-grade facilities across Africa. Compliance with Good Manufacturing Processes (GMP) is non-negotiable, yet many existing facilities on the continent fall short of WHO prequalification standards. In addition, Africa currently lacks a sufficient pool of trained specialists in bioprocess engineering, Quality Assurance/Quality Control, cleanroom operations, and GMP documentation. The AU’s goal of training over 15,000 biospecialists by 2030 will require the development of a comprehensive curriculum, international fellowships, and incentives to retain skilled professionals in the region. Finally, advancing the vaccine ecosystem in Africa will also require access to affordable, high-quality raw materials and consumables that are currently limited and the transfer of complex processes for upstream and downstream bioprocessing, formulation, quality assurance and quality control, and regulatory documentation.

What is intended to be the first scaled-up vaccine that is manufactured on the African continent?

The 2024 Africa CDC landscape report, developed with CHAI and PATH, confirms significant progress toward vaccine self-reliance: 25 active vaccine projects are underway across the continent. Crucially, three manufacturers are projected to achieve WHO prequalification for nine vaccine products between 2026–2030; eight antigens are expected to achieve WHO PQ and enter the continental market between 2025-2030: Senegal (Yellow Fever and MR) and South Africa (Hexa, PCV, MMCV, Rota, OCV and IPV).

This geographically diversified pipeline spanning Senegal (West Africa), and South Africa (Southern Africa) directly advances regional cooperation by distributing manufacturing capacity across key hubs. It simultaneously builds local capability through technology transfer, workforce development, and regulatory alignment under PAVM framework for action.

The first vaccine after Yellow Fever, expected to be manufactured at scale on the African continent, is the simplified oral cholera vaccine. Production is expected to ramp up significantly by 2028, positioning this vaccine as the first major output of Africa’s scaled-up manufacturing efforts. Other candidates for early scale-up include hexavalent vaccines, Rotavirus, Polio, Measles-Rubella, and malaria vaccines, particularly as partnerships deepen on technology transfer with multinational companies.

Nicaise, what are the main focuses of your current role at the IVI?

My current role has a big focus on fostering ethical, regulatory, human capital development, and operational readiness to help Africa become a leader, and not just a beneficiary, in global vaccine development. I coordinate cooperation at the political, international, regional, national, and public-private interface levels. There are two main areas of focus, which are to build and develop Africa’s human professional capacity and research and development capacity for clinical trials.

An estimated 5.5 million professionals will be needed in Africa to help achieve the AU’s goal of localizing 60% of vaccine manufacturing by 2040. These professionals will work across all aspects of the process in surveillance ( < 3,000), clinical research (6,000), regulatory (10,000), manufacturing (18,000), supply chain (200,000), and service delivery (5 million). As part of my role at IVI, I develop capacity-building programs for attracting and retaining scientific talent in Africa^10^.

Additionally, I lead efforts to design and implement context-relevant clinical trials ecosystems and networks across the continent to support endemic and emerging infectious disease research. The achievement of the oral cholera vaccine (OCV-S) technology transfer to Biovac in South Africa by the IVI is just one example of efforts to strengthen research and development and vaccine manufacturing capacity.

What are the remaining main development efforts, interventions and investment requirements that are left to achieve the goal of greater African self-sufficiency in vaccine manufacturing and capacity building?

Africa’s aims for health security are not aspirational dreams; they are achievable imperatives, which are grounded in policy, science, innovation and the urgent lessons from the COVID-19 pandemic. The continent has made significant strides in developing policy frameworks, such as the AU’s Roadmap to 2030, and developing manufacturing hubs and regulatory institutions like the African Medicines Agency.

There is still work to be done. Africa must invest in supply chain and logistics infrastructure, including cold chain systems and raw material production. Currently, most of the inputs for vaccine manufacturing are imported, and developing upstream capabilities such as active pharmaceutical ingredients (APIs) and packaging materials is critical for long-term resilience. Additionally, important remaining interventions include expanding fill-and-finish capabilities, securing long-term procurement commitments, and strengthening regulatory harmonization across regional economic communities. Investment in workforce development, including biotechnology training programs, regional centers of excellence, and STEM education pipelines, is vital to ensure a sustainable talent base.

Finally, political commitment must be matched with regional coordination mechanisms to avoid duplication, ensure equitable distribution, and enable knowledge-sharing across countries and manufacturing nodes. Alignment between the Africa CDC, AUDA NEPAD, AMA, RECs, and national regulatory authorities will be essential to ensure that efforts remain synergistic and scalable.
