# Publisher Correction: Strengthening vaccine capacity building on the African continent

**DOI:** 10.1038/s41467-025-65106-2

**Published:** 2026-02-12

**Authors:** Nicaise Ndembi, Jerome H. Kim

**Affiliations:** 1 International Vaccine Institute (IVI), Norrsken House, 1 KN 78 St, Kigali, Rwanda; 2 International Vaccine Institute (IVI), College of Natural Sciences, Seoul Natural University, Seoul, Republic of Korea

Correction to: *Nature Communications* 10.1038/s41467-025-62839-y, published online 13 August 2025

In the version of the article initially published, the authors Nicaise Ndembi (International Vaccine Institute, Kigali, Rwanda) and Jerome Kim (International Vaccine Institute, College of Natural Sciences, Seoul Natural University, Seoul, Republic of Korea) were not displayed in the article and are now included in the HTML and PDF versions of the article. The copyright holder for this article was incorrectly given as ‘Springer Nature Limited’ but should have been ‘The Author(s)’. The original article has been corrected.

